# Impact of smoking and alcohol drinking on the prognosis of 721 nasopharyngeal carcinoma

**DOI:** 10.1016/j.bjorl.2024.101534

**Published:** 2024-11-19

**Authors:** Ling Tian, Min Zhao, Qing Yang, Xiaojiang Li, Yun Chen, Wu Xifang, Yan-Xin Ren

**Affiliations:** a Third Affiliated Hospital of Kunming Medical University, Head and Neck Surgery Department, Kunming, Yunnan, China; b Third Affiliated Hospital of Kunming Medical University, Cancer Prevention and Control Department, Kunming, Yunnan, China; c Third Affiliated Hospital of Kunming Medical University, Pathology Department, Kunming, Yunnan, China

**Keywords:** Nasopharyngeal carcinoma, Prognostic factor, Smoking, Alcohol drinking

## Abstract

**Objective:**

This study aimed to examine the correlation between smoking, alcohol drinking, and the prognosis of Nasopharyngeal Carcinoma (NPC).

**Methods:**

Clinical data from 721 NPC cases treated at our hospital between January 2005 and December 2010 were collected. Information on smoking and drinking, including duration, daily quantity, and cumulative amount, was recorded and graded according to WHO standards. Statistical analysis was performed to assess the influence of smoking and alcohol drinking on NPC patient prognosis. After controlling for confounding factors, survival analysis compared the 5-year Progression-Free Survival rate (PFS) and Overall Survival rate (OS) among patients with varying degrees of smoking and drinking. The association between smoking, drinking, cumulative amount, and NPC patient prognosis was evaluated. Multivariate Cox regression analysis was then employed, considering patient demographic characteristics and clinical features, to comprehensively analyze prognostic influencing factors in NPC patients. Additionally, the multivariate Cox regression analysis was utilized to comprehensively examine the influencing factors of prognosis, taking into account the patients' basic demographic characteristics and clinical features. The findings revealed significant differences in the aforementioned rates.

**Results:**

(1) Analysis of PFS and OS differences in NPC patients considered smoking status, smoking duration, daily smoking quantity, and cumulative smoking amount. No significant influence of smoking on NPC patient PFS and OS within 5-years was observed (*p* > 0.05). (2) Non-drinkers with NPC exhibited higher 5-year PFS and OS rates compared to drinkers (*p* = 0.047, *p* = 0.026). Furthermore, non-drinkers and those with a drinking duration of less than 120 months or between 120–240 months showed higher 5-year PFS and OS rates than individuals with a drinking duration exceeding 240 months (*p* < 0.05). Similarly, non-drinkers and individuals consuming less than 50 g/day had higher 5-year PFS and OS rates compared to those consuming 50–100 g/day or more than 100 g/day (*p* < 0.05). Additionally, the 5-year PFS and OS rates were higher in the non-drinking and light drinking groups compared to the moderate and heavy drinking groups (*p* < 0.05). (3) A partial synergistic effect between smoking and alcohol drinking was observed in NPC. (4) Alcohol drinking emerged as an independent prognostic factor for NPC.

**Conclusion:**

Alcohol drinking is a significant factor influencing the prognosis of nasopharyngeal carcinoma, with the adverse effects further amplified when combined with smoking.

**Level of evidence:**

Level 2.

## Highlights

• Heavy alcohol drinking is an independent prognosis factor for NPC in Yunnan Province.
• NPC patients who smoke and drink alcohol at the same time had a poorer prognosis.
• NPC patients should be advised to quit smoking and alcohol for better prognosis.

## Introduction

Nasopharyngeal Carcinoma (NPC) is a malignant tumor originating from the epithelial cells of the nasopharynx. NPC has high incidence rates in a few specific populations such as Hong Kong and southern China, especially in the Pearl River Delta region with an incidence rate ranging from 40 to 50 cases per 100,000 individuals.^1^ The important step to prevent NPC is to identify and understand factors that contribute to this large discrepancy in NPC rates. Epstein-Barr Virus (EBV) may be a critical etiological factor for development of NPC.^2^ However, a large proportion of individuals worldwide are infected with the virus, but only a small proportion of them develop NPC.

While genetic and environmental play an important role in NPC carcinogenesis,^3^ lifestyle factors, such as tobacco smoking and alcohol drinking are established risk factors for NPC,^4^ these behaviors have been recognized as significant contributors to the development of NPC, potentially influencing patient prognosis.^5^ However, there is conflicting evidence regarding the specific prognostic value of smoking and drinking in NPC. Some studies suggest that smoking is considered a causative factor for NPC, with heavy smokers exhibiting a 2–4 times increased risk of developing the disease. Nevertheless, other studies fail to establish a clear correlation between smoking and NPC development.^6^ The two different NPC cohort studies conducted in low-risk areas, one from the US reported a significant trend with daily smoking amount, but this study did not find a significant trend for smoking duration and did not report results on cumulative consumption. The other study from the British cohort did not report any significant results. Conversely, while drinking is not found to be a causative factor for NPC among Chinese populations, it has been identified as a risk factor for NPC in Western populations. It is possible that alcohol drinking does not contribute to NPC risk, but other potential reasons for the inconsistencies are differences in study design, geographic location, different histopathology, measurement methods for alcohol intake, and adjustment of other risk factors for NPC across studies. This inconsistency has led to controversy surrounding the specific prognostic implications of smoking and drinking in NPC patients.^7^ In order to provide more scientific evidence on the prognostic effects of smoking and drinking in NPC, a systematic retrospective study was conducted involving 721 NPC patients in Yunnan province, China.

## Methods

### Research subjects

A retrospective review was conducted of NPC patients treated at Third Affiliated Hospital of Kunming Medical University between January 1, 2005, and December 31, 2010.Inclusion criteria were: (1) Newly diagnosed, histologically proven NPC; (2) Without distant metastasis; (3) Receiving radical radiotherapy. Exclusion criteria included patients were: (1) Younger than 18 years old; (2) Lack of the record of alcohol intake habits; (3) With any history of malignancy or immunologically mediated disease cardiovascular diseases, respiratory diseases. Thus, the remaining 721 NPC patients were enrolled in this study, including 535 male cases and 186 female cases, ranging in age from 11 to 89 years, with a median age of 47 years. Computed Tomography (CT) and/or Magnetic Resonance Imaging (MRI) examinations were essential for all patients, and the staging were recorded according to the 7th edition of the Union for International Cancer Control/American Joint Committee on Cancer (UICC/AJCC) staging system for NPC.

### Treatment methods

Based on the 2020 edition of the National Comprehensive Cancer Network (NCCN) guidelines, patients in stage I received radical radiotherapy, while patients in stages II, III, and IV without distant metastases received radiotherapy combined with chemotherapy. All radiotherapy treatments were conducted using Three-Dimensional Conformal Radiotherapy (3D-CRT). The dose administered in the nasopharyngeal target area ranged from 68 to 75 Gy in 29–35 fractions, while the cervical lymph node drainage area received a dose of 60–65 Gy in 25–30 fractions. Among the 60 patients with distant metastases, 19 received palliative radiotherapy. Among the 704 cases where chemotherapy was administered, 430 underwent induction chemotherapy + concurrent chemoradiotherapy, 233 received concurrent chemoradiotherapy + adjuvant chemotherapy, and 41 underwent chemotherapy alone. The main chemotherapy regimens consisted of cisplatin 80 mg/m^2^ (day 1–3) + fluorouracil 500–750 mg/m^2^ (day 1–5), and docetaxel 75 mg/m^2^ (day 1) + cisplatin 80 mg/m^2^ (day 1–3), with each cycle lasting 21 days. For concurrent chemotherapy, cisplatin 100 mg/m^2^ (day 1–3) alone or in combination with fluorouracil 500–750 mg/m^2^ (day 1–5) was administered for 21 days per cycle.

### Clinical information collection

The information of patients ‘alcohol intake, smoking, and specific lifestyle habits were collected. The primary outcome measure considered was Overall Survival (OS), defined as the time from start of treatment to either death from any cause or the last follow-up visit. The secondary outcome measure was disease-Free Progression Survival (PFS), defined as the time from start of treatment to disease progression or death due to nasopharyngeal carcinoma.

### Patient grouping criteria

Smoking status was determined according to the World Health Organization's definition of smokers as individuals over 20 years old who smoke at least one cigarette per day for 6-months or longer, or adolescents aged 15–19 years who smoke at least one cigarette per week for 3-months or longer. Based on the duration of smoking, patients were categorized into non-smoking, <120 months, 120–240 months, and >240 months groups.^8^ According to the amount of smoking per day, patients were assigned to non-smoking, <10 cigarettes/day, 10–20 cigarettes/day, and >20 cigarettes/day groups.^9^ Moreover, the cumulative amount of smoking (number of cigarettes per day × smoking years) led to the division into non-smoking, light smoking (<300 cigarettes), moderate smoking (300–600 cigarettes), and heavy smoking (>600 cigarettes) groups.^5^ Regarding alcohol drinking, patients were divided based on the duration of alcohol drinking into no-drinking, <120 months, 120–240 months, and >240 months groups. Alcohol drinking per day resulted in groupings as follows: no-drinking, <50 g/day, 50–100 g/day, and >100 g/day groups.^10^ Finally, the cumulative amount of alcohol drinking (grams of alcohol per day × drinking years) led to categorization into no-drinking, light drinking (≤1000 g), moderate drinking (1000–3000 g), and heavy drinking (≥3000 g) groups.^9^

### Observation and follow-up

After completion of treatment, patients were followed up every 3-months for the first 3-years and the intervals gradually increased to 6–12 months after 3-years. The follow-up included routine blood tests, blood biochemistry, CT or MRI scans of the nasopharynx, chest X-Ray or CT, neck and abdominal ultrasound, isotope bone scan, and nasopharyngoscopy. Detailed results were recorded during each visit.

### Statistical methods

Statistical analysis was performed using SPSS 19.0 software. Count data were expressed as n (%), and the chi-square test was employed for between-group comparisons. The rates of OS, PFS were estimated by means of the Kaplan-Meier method and differences were compared using the log-rank test. Multivariate analysis using a Cox proportional hazards model was used to test the independent significance of different variables by enter method of insignificant explanatory variables. The covariates entering into the multivariable analysis included host factors (age), tumor factors (T and N classification), smoking status, and smoking index.

## Results

### Case characteristics

The baseline characteristics of the whole patients analyzed in this study are shown in Table 1. Among the 721 patients, 343 individuals (47.6%) were smokers, and their smoking status exhibited associations with age and gender (p < 0.05). However, no significant associations were observed between smoking status and ethnicity, pathological type, stage, lymph node metastasis, distant metastasis, or EBV infection (*p* > 0.05). Additionally, 166 patients (23%) reported alcohol drinking, which also showed associations with age and gender (*p* < 0.05). Similar to smoking, no significant correlations were found between alcohol drinking and ethnicity, pathological type, stage, lymph node metastasis, distant metastasis, or EBV infection (*p* > 0.05). The amount of smoking and drinking per day, as well as the cumulative smoking and drinking, demonstrated statistically significant differences within the overall population (*p* < 0.001). Among the 721 patients, a 5-year follow-up period revealed that 145 (20.1%) were lost to follow-up, 65 (9.0%) developed locoregional failure, 60 (8.32%) developed distant metastasis, and 173 (23.9%) died. For the entire cohort, the 5-year PFS, OS rates were 76.0% and 78.0%, respectively.

**Table 1:** Clinical Characteristics of NPC.

	Smoking		χ^2^	*p*	Drinking		χ^2^	p
	Yes	No			Yes	No		
Age								
≤30	18 (33.3%)	36 (66.7%)	**13.36**	**0.01**	2 (3.7%)	52 (96.3%)	**20.403**	**<0.001**
31‒40	60 (40%)	90 (60%)			25 (16.7%)	125 (83.3%)		
41‒50	121 (54.5%)	101 (45.5%)			65 (29.3%)	157 (70.7%)		
51‒60	97 (46.9%)	11 (53.1%)			52 (25.1%)	155 (74.9%)		
≥61	47 (53.4%)	41 (46.6%)			22 (25%)	66 (75%)		
**Sex**								
Male	343 (64.1%)	192 (35.9%)	**227.456**	**<0.001**	165 (30.8%)	370 (69.2%)	**71.513**	**<0.001**
Female	0 (0%)	186 (100%)			1 (0.5%)	185 (99.5%)		
**Nationalities**								
Han	302 (48.5%)	321 (51.5%)	9.79	0.08	138 (22.2%)	485 (77.8%)	7.297	0.199
Yi	15 (48.4%)	16 (51.6%)			11 (35.5%)	20 (64.5%)		
Bai	8 (53.3%)	7 (46.7%)			3 (20%)	12 (80%)		
Hani	7 (70%)	3 (30%)			5 (50%)	5 (50%)		
Dai	1 (16.7%)	5 (83.3%)			1 (16.7%)	5 (83.3%)		
Others	10 (28.6%)	25 (71.4%)			8 (22.9%)	27 (77.1%)		
**Type of pathology**								
NKSC	314 (48.2%)	337 (51.8%)	2.59	0.274	147 (22.6%)	504 (77.4%)	1.349	0.509
KSC	29 (42.6%)	39 (57.4%)			18 (26.5%)	50 (73.5%)		
BCSC	0 (0%)	2 (100%)			1 (50%)	1 (50%)		
**TNM Staging**								
Ⅰ+Ⅱ	81 (45.5%)	97 (54.5%)	0.611	0.559	41 (23%)	137 (77%)	0.710	0.991
Ⅲ+Ⅳ	256 (48%)	277 (52%)			123 (23.1%)	410 (76.9%)		
**Lymph node metastasis**								
Yes	286 (48.1%)	309 (51.9%)	2.022	0.563	139 (23.4%)	456 (76.6%)	1.962	0.64
No	57 (45.2%)	69 (54.8%)			27 (21.4%)	99 (78.6%)		
**Distant transfer**								
Yes	29 (48.3%)	31 (51.7%)	0.015	0.902	16 (26.7%)	44 (73.3%)	0.490	0.484
No	314 (47.5%)	347 (52.5%)			150 (22.7%)	511 (77.3%)		
**EBV‒DNA**								
Positive	122 (47.6%)	134 (52.4%)	0.027	0.870	55 (21.4%)	201 (78.6%)	0.002	0.962
Negative	214 (46.1%)	250 (53.9%)			98 (21.1%)	366 (78.9%)		
Cigarettes smoked/day								
0	0 (0%)	378 (100%)			19 (5.0%)	358 (95.0%)		
<10	130 (100%)	0 (0%)	‒	‒	18 (51.4%)	17 (48.6%)	145.226	**<0.001**
10‒20	166 (100%)	0 (0%)			110 (42.6%)	148 (57.4%)		
>20	47 (100%)	0 (0%)			20 (39.2%)	31(60.8%)		
Cumulative amount of smoking								
No	0 (0%)	378 (100%)			19 (5.0%)	358(95.0%)		
Light	35 (100%)	0 (0%)	‒	‒	47 (36.2%)	83 (63.8%)	150.656	**<0.001**
Moderate	257 (100%)	0 (0%)			77 (46.4%)	89 (53.6%)		
Heavy	51 (100%)	0 (0%)			23 (48.9%)	24 (51.1%)		
Amount of drink/day								
0	196 (35.3%)	359 (64.7%)			0(0%)	555(100%)		
<50 g	40 (90.9%)	4 (9.1%)	145.481	**<0.001**	44 (100%)	0 (0%)	‒	‒
50‒100 g	51 (89.5%)	6 (10.5%)			57 (100%)	0 (0%)		
> 100 g	56 (86.2%)	9 (13.8%)			65 (100%)	0 (0%)		
Cumulative amount of drinking								
No	196 (35.3%)	359 (64.7%)			0 (0%)	555 (100%)		
Light	40 (90.9%)	4 (9.1%)	145.481	**<0.001**	44 (100%)	0 (0%)	‒	‒
Moderate	51 (89.5%)	6 (10.5%)			57 (100%)	0 (0%)		
Heavy	56 (86.2%)	9 (13.8%)			65 (100%)	0 (0%)		

NKSC, Non-Keratinizing Squamous Carcinoma; KSC, Keratinizing Squamous Carcinoma; BCSC, Basal Cell-Like Squamous Carcinoma.

### Influence of smoking on NPC prognosis

**Fig. 1:**
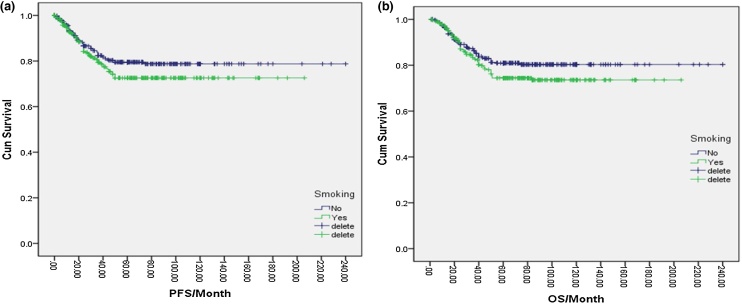
(a) Disease free survival curve of NPC according to smoking or not. (b) Overall survival curve of NPC according to smoking or not.

**Fig. 2:**
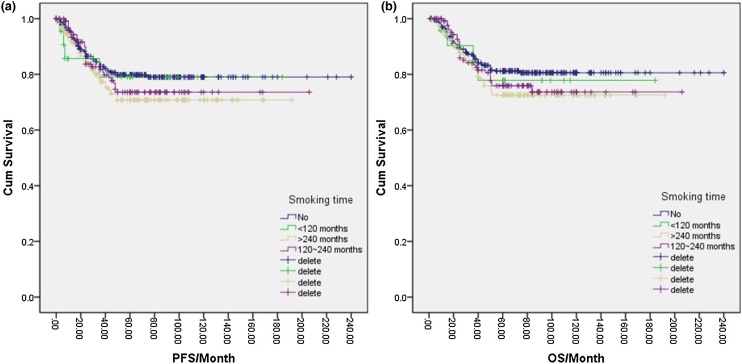
(a) Disease free survival curve of NPC according to smoking time. (b) Overall survival curve of NPC according to smoking time.

**Fig. 3:**
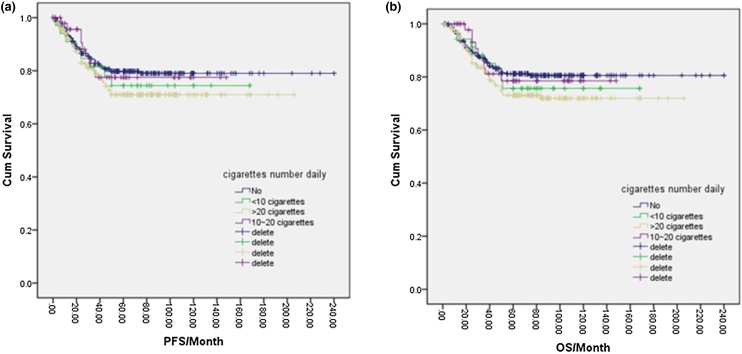
(a) Disease free survival curve of NPC according to cigarettes number daily. (b) Overall survival curve of NPC according to cigarettes number daily.

**Fig. 4:**
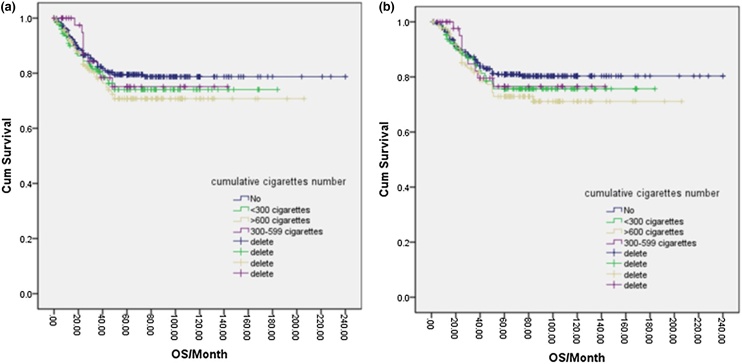
(a) Disease free survival curve of NPC according to cumulative cigarettes number. (b) Overall survival curve of NPC according to cumulative cigarettes number.

Of the total NPC cases in this study, 343 (47.6%) were smokers, while 378 (52.4%) were complete nonsmokers. One-way logistic regression analysis indicated that the 5-year PFS and OS rates for nonsmokers were 79.0% and 80.0%, respectively, compared to 72.0% and 74.0% for smokers. However, these differences were not statistically significant (*p* = 0.094, *p* = 0.077) (Fig. 1a, b). Further analysis of various smoking factors and their effects on NPC prognosis revealed no significant differences in PFS or OS among the nonsmoking group, <120-months group, 120–240 months group, and >240-months group based on smoking duration (*p* = 0.279, **p** = 0.251) (Fig. 2a, b). Similarly, no significant differences were found in PFS or OS between the nonsmoking, <10 cigarettes/day, 10–20 cigarettes/day, and >20 cigarettes/day groups based on the amount of cigarettes smoked per day (*p* = 0.238) (Fig. 3a, b). Finally, the cumulative amount of smoking did not demonstrate any significant associations with PFS or OS when comparing the nonsmoking, light smoking, moderate smoking, and heavy smoking groups (*p* = 0.322) (Fig. 4a, b). Overall, there was no significant influence of smoking on the prognosis of NPC patients.

### Influence of alcohol intake on NPC prognosis

**Fig. 5:**
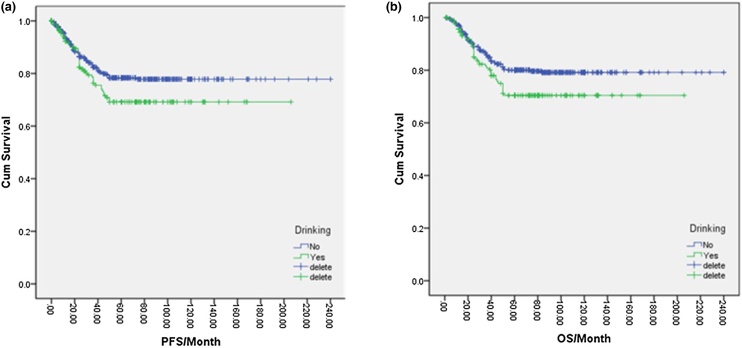
(a) Disease free survival curve of NPC according to alcohol drinking or not. (b) Overall survival curve of NPC according to alcohol drinking or not.

**Fig. 6:**
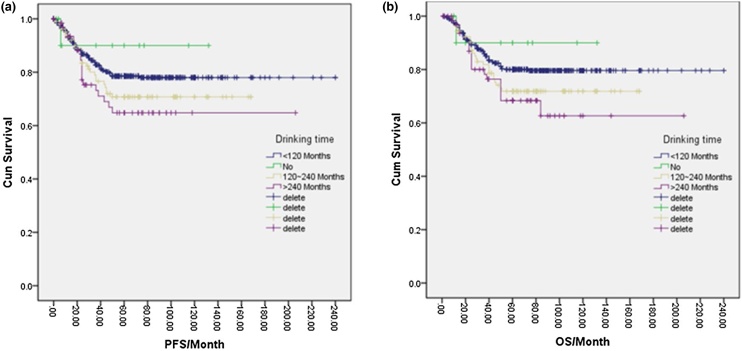
(a) Disease free survival curve of NPC according to alcohol drinking time. (b) Overall survival curve of NPC according to alcohol drinking time.

**Fig. 7:**
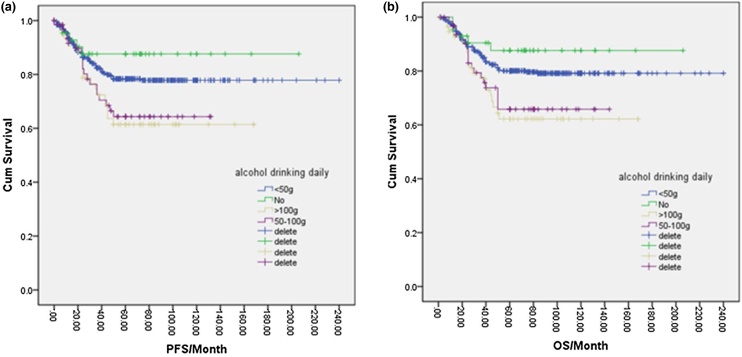
(a) Disease free survival curve of NPC according to alcohol drinking daily. (b) Overall survival curve of NPC according to alcohol drinking daily.

**Fig. 8:**
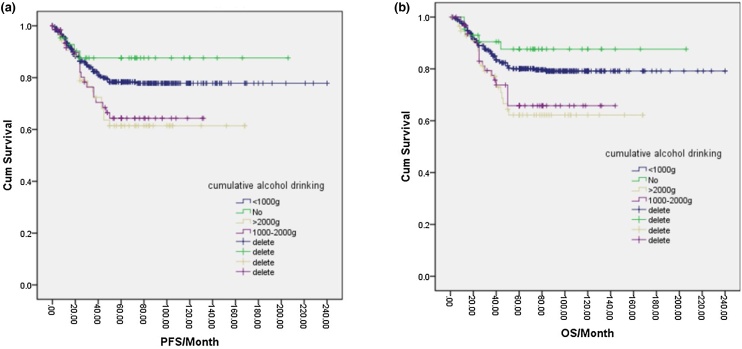
(a) Disease free survival curve of NPC according to cumulative alcohol drinking. (b) Overall survival curve of NPC according to cumulative alcohol drinking.

In total, 166 patients (23%) were drinkers, while 555 patients (77%) were non-drinkers. One-way logistic regression analysis indicated that the 5-year PFS for non-drinkers was 78.0%, which was higher than the 69.0% observed in drinkers, and the difference was statistically significant (*p* = 0.047). Additionally, the 5-year OS for non-drinkers was 80.0%, compared to 70.0% in drinkers, with a significant difference (*p* = 0.026) (Fig. 5a, b). Further analysis of various drinking factors and their effects on NPC prognosis revealed significant differences in 5-year PFS and OS between the no-drinking group, <120-month group, 120–240 month group, and >240-month group (*p* = 0.021, *p* = 0.035, and *p* = 0.038). Furthermore, the 5-year OS was higher in the no-drinking group, <120-month group, and 120–240-month group compared to the >240-month group (*p* = 0.015, *p* = 0.018, and *p* = 0.026) (Fig. 6a, b). Similarly, the 5-year PFS and OS differed significantly among the no-drink group, <50 g/day group, 50–100 g/day group, and >100 g/day group based on the amount of alcohol intake per day (*p* = 0.03, *p* = 0.041, *p* = 0.01, *p* = 0.018). Higher 5-year OS rates were observed in the no-drink group and <50 g/day group compared to the 50–100 g/day group and > 100 g/day group (*p* = 0.012, *p* = 0.043, *p* = 0.02, *p* = 0.046) (Fig. 7a, 7b). Likewise, the cumulative amount of drinking resulted in distinct differences in 5-year PFS and OS between the no-drinking group and light-drinking group compared to the moderate-drinking group and heavy-drinking group (*p* = 0.03, *p* = 0.032, *p* = 0.02, *p* = 0.025) (Fig. 8a, b). These results indicated that longer durations of drinking and higher daily alcohol intake were unfavorable prognostic factors for NPC patients.

### Synergistic relationship between smoking and drinking

**Fig. 9:**
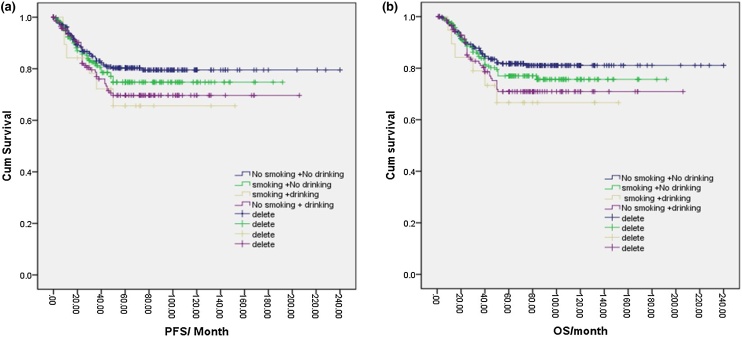
(a) Disease free survival curve of NPC according to smoking and alcohol drinking. (b) Overall survival curve of NPC according to smoking and alcohol drinking.

Based on smoking and drinking status, patients were categorized into the non-smoking and non-drinking group (358 cases, 49.6%), smoking and non-drinking group (196 cases, 27.2%), drinking and non-smoking group (20 cases, 2.8%), and smoking and drinking group (147 cases, 20.4%). The 5-year PFS and OS rates for these groups were 80.0%, 75.0%, 69.0%, 66.0% and 81.0%, 77.0%, 71.0%, 66.0%, respectively. Notably, the nonsmoking and nondrinking group exhibited higher PFS and OS rates compared to the smoking and drinking group (*p* = 0.045, *p* = 0.004), while no statistical differences were observed among the other groups comparisons (*p* = 0.151, *p* = 0.091) (Fig. 9a, b).

### Multivariate analysis of smoking and drinking as independent prognostic factors for NPC

Multifactorial regression analysis was conducted to evaluate various clinical characteristics, including pathological type, age at presentation, ethnicity, gender, TNM stage, N stage, T stage, smoking status, and alcohol intake, as potential independent prognostic factors for NPC. The analysis indicated that smoking status, amount of smoking per day, and cumulative amount of smoking were not independent prognostic factors for NPC (*p* = 0.419, *p* = 0.900, *p* = 0.905). However, alcohol intake and the cumulative amount of drinking emerged as independent prognostic factors for NPC (*p* = 0.046, *p* = 0.043) (Table 2).

**Table 2:** Multivariate Cox regression analysis for the patients with NPC.

	B	SE	Wald	*p*	HR	95% CI	
Age	0.102	0.042	5.976	**0.015**	1.108	1.021	1.203
Sex	0.081	0.115	0.490	0.484	1.084	0.865	1.358
TNM Staging	0.366	0.108	11.535	**0.001**	1.442	1.167	1.781
Smoking or not	−0.298	0.369	0.652	0.419	0.742	0.360	1.530
Drinking alcohol or not	−0.385	0.305	4.5645	**0.046**	0.566	0.208	1.015
Drinking time	0.124	0.169	0.535	0.465	1.132	0.812	1.577
Amount of drinking per day	0.341	0.205	2.775	0.096	1.407	0.942	2.102
Cumulative amount of drinking	0.406	0.201	4.089	**0.043**	1.501	1.013	2.226
Smoking and drinking	0.160	0.068	5.606	**0.018**	1.174	1.028	1.340

## Discussion

Some lifestyle behaviors are established risk factors and potential prognostic factors for NPC. To date, the intricate composition of cigarette smoke has been extensively scrutinized, leading to the identification of over 70 known carcinogens. Carcinogenic substances within cigarette have the capability to bind to DNA and disrupt the delicate structure of the double helix, resulting in the formation of DNA adducts. If not repaired, these DNA adducts can instigate coding errors and permanent mutations that either activate oncogenes or deactivate tumor suppressor genes.^11^ Remarkably, the investigation conducted by the authors revealed substantial levels of DNA adducts released by constituents of cigarettes in the buccal mucosa of individuals afflicted with head and neck squamous carcinoma, providing suggestive evidence of an association between cigarette smoke components and the development of tumors.^12^ Further inquiry elucidated that extracts derived from cigarette foster the replication of EBV and prompt the expression of immediate early transcriptional activators in vitro, subsequently leading to enhanced transcription of BFRF3 and gp350 during cleavage. These consequential outcomes could potentially account for the heightened prognostic significance of smoking status in individuals deemed to be at a heightened risk for nasopharyngeal carcinoma.^13^

Notably, studies have corroborated the detrimental impact of smoking on male patients with nasopharyngeal carcinoma receiving radiation therapy, showcasing unfavorable prognostic implications.^14^ In terms of the patient's survival outcome, it was observed that discontinuing smoking did not yield any significant changes. Notably, in male individuals who were heavy smokers, the risk of mortality was found to be 1.225 times higher compared to those with lower smoking intensity. Subsequent analysis unveiled that the prognostic influence of smoking volume predominantly stemmed from cases of extensive smoking, indicated by a smoking index surpassing 45 packs per year. As the smoking index escalates, the risk of death in male patients diagnosed with nasopharyngeal carcinoma exhibits a corresponding increase.^15^ Additionally, Chen AM et al. conducted a meticulously controlled investigation involving patients afflicted with head and neck squamous carcinoma who underwent radiation therapy. The findings demonstrated that individuals who persisted in smoking throughout the course of radiation treatment exhibited significantly inferior 5-year overall survival rates, local area control, and disease-free survival when compared to their non-smoking counterparts.^16^ Moreover, Lv JW et al. unraveled that smoking exerts an impact on Overall Survival (OS) and Progression-Free Survival (PFS) in patients with nasopharyngeal carcinoma. Notably, cut-off points for OS and PFS were established at 32 pack-years and 22 pack-years, respectively.^13^

Two hypotheses have been proposed to elucidate the potential mechanisms underlying the adverse prognosis observed in smokers diagnosed with NPC. Firstly, one hypothesis implicates hypoxia as a contributing factor. Smoking has been correlated with heightened levels of carboxyhemoglobin in the bloodstream, thereby causing a leftward shift in the hemoglobin-oxygen dissociation curve.^17^ Consequently, this elevation in carboxyhemoglobin among smokers may account for the negative impact on the local control achieved through radiotherapy. The presence of increased carboxyhemoglobin diminishes the oxygen supply to the tumor, ultimately compromising the efficacy of radiation therapy.^18^ Alternatively, another plausible mechanism could be attributed to the modulation of Natural Killer (NK) cells. Smoking has been found to impede the activation of NK cells and attenuate the Cytotoxic Lymphocyte (CTL) activity exerted by these immune cells. This impairment can subsequently alter the tumor microenvironment, potentially expediting tumor progression, metastasis, and augmenting the overall tumor burden experienced by individuals with NPC.^19^ These two proposed mechanisms shed light on the multifaceted nature of the deleterious effects exerted by smoking on the prognosis of patients diagnosed with NPC. By influencing factors such as hypoxia and NK cell functionality, smoking appears to intricately impact various aspects of the disease's pathogenesis and progression. Further research is warranted to comprehensively understand these intricate mechanisms and devise strategies to mitigate their detrimental consequences in clinical practice.

In our study cohort, no statistically significant disparities were observed in OS and PFS when comparing participants based on their smoking status as a whole. However, upon conducting further stratification analyses considering both the daily quantity of cigarettes smoked and the duration of smoking, noteworthy distinctions emerged. Specifically, individuals characterized as long-term smokers and heavy smokers, with a substantial daily cigarette consumption, exhibited lower OS and PFS rates compared to non-smokers, as well as light and moderate smokers. These findings corroborate previous investigations that have reported similar outcomes. It is important to acknowledge certain limitations inherent in our study, including the absence of detailed information regarding variables such as the age at which patients-initiated smoking, the specific type of tobacco used, and the mode of smoking employed. The lack of this granular data may have influenced the statistical results obtained, necessitating caution when interpreting these findings. Future research endeavors should aim to address these limitations by incorporating comprehensive data collection protocols to enhance the precision and robustness of subsequent statistical analyses.

The impact of alcohol intake on the prognosis of NPC remains a subject of debate. However, several lines of evidence substantiate the biologically plausible connection between heavy drinking and an increased risk of NPC development, aligning with the observed association between alcohol intake and heightened susceptibility to upper respiratory and gastrointestinal cancers. Ethanol, a prominent constituent of alcoholic beverages, is considered the key compound underlying the influence of alcohol drinking on tumor progression. Of particular significance is acetaldehyde, an oxidation product of alcohol that exhibits toxic, carcinogenic, and mutagenic properties. Experimental and animal studies have demonstrated that acetaldehyde interferes with DNA synthesis and repair at multiple loci, potentially contributing to tumorigenesis. Furthermore, chronic alcohol drinking induces the activity of cytochrome P450 enzymes, specifically CYP2E1, in mucosal cells. This enzymatic induction promotes the formation of free radicals, triggering cellular damage.^20^ Additionally, chronic heavy drinking can give rise to deficiencies in various essential vitamins and trace elements, including folic acid, iron, zinc, and vitamin A. These micronutrient deficiencies are intricately involved in gene regulation and cell differentiation processes.^21^ Chen YP et al.^22^ conducted a study revealing lower 5-year OS rates and local recurrence-free survival rates among drinkers compared to non-drinkers in NPC patients. Moreover, drinking ≥14 drinks per week and having a drinking history of ≥ 20 years emerged as independent adverse prognostic factors for OS. However, no significant differences were observed between light drinkers and non-drinkers. Several mechanisms may underlie the influence of alcohol drinking on the prognosis of NPC. Firstly, alcoholics tend to consume less food containing essential nutrients, and alcohol, along with its metabolites, may further impede the absorption and utilization of these vital nutrients, thereby inducing malnutrition.^23^ Secondly, alcohol drinking impairs immune surveillance and clearance mechanisms through altered cytokine production, aberrant reactive oxygen species generation, diminished NK cell activity, and compromised cell-mediated immunity.^24^ Thirdly, miRNA expression patterns were found to be altered in patients with head and neck squamous carcinoma who consumed alcohol, with high miR-21 expression associated with significantly lower 5-year survival rates.^25^ These findings collectively highlight the multifaceted ways in which alcohol drinking may impact the prognosis of NPC. The intricate interplay between nutrient deficiencies, immune dysfunction, and altered molecular pathways underscores the need for comprehensive investigations to further elucidate these mechanisms in order to optimize patient management strategies.

However, an intriguing study conducted by Kampa M et al.^26^ presented contrasting findings in a meta-analysis of drinking dosage and the risk of NPC. Their analysis revealed that moderate alcohol intake exhibited a lower risk compared to no alcohol intake or higher levels of consumption. Similarly, Li A.D. et al.^27^ observed a protective effect of small amounts of alcohol drinking in patients with NPC. Moreover, another investigation indicated that specific types of alcohol had divergent impacts on NPC incidence. Specifically, red wine and Chinese yellow wine were associated with reduced NPC risk, whereas spirits and beer were linked to increased incidence rates.^28^ The underlying mechanism driving these effects may be attributable to the relatively high concentrations of polyphenols, particularly flavonoids and resveratrol, present in wine. These compounds possess potential antioxidant and chemopreventive properties, thereby inhibiting the development, promotion, and progression of cancer. Furthermore, it is noteworthy that the impact of alcohol drinking on NPC risk varies among different ethnic groups. Pan et al.^29^ analyzed the stronger association between alcohol drinking and NPC development within the United States population. This association was found to be influenced by the pathological type of NPC as well as the timing of EBV infection in relation to alcohol drinking. In the United States, the NPC population is predominantly characterized by keratinized squamous cell carcinoma, which exhibits a closer relationship with alcohol drinking. Conversely, NPC cases in China are mainly classified as non-keratinized squamous and undifferentiated carcinoma. On the topic of long-term alcohol intake, initiation age of drinking, abstinence from alcohol, and their associations with NPC, Du T et al.^7^ reported no significant associations. However, consumption exceeding seven drinks per week was associated with an increased NPC risk, while consuming less than seven drinks per week showed a protective effect compared to abstaining from alcohol. Feng R et al.^30^ also found no direct correlation between alcohol intake and NPC. Additionally, another study reported a J-shaped dose-response trend, indicating that light or moderate alcohol drinkers had a significantly or non-significantly lower risk of NPC, while heavy drinkers exhibited an increased risk of the disease.^31^ These diverse findings underscore the complex relationship between alcohol drinking and NPC risk. Factors such as the dosage and type of alcohol, ethnic disparities, and potential interactions with other variables can influence the observed associations. Further investigations are warranted to comprehensively understand these intricacies and provide clearer insights into the role of alcohol in NPC pathogenesis and prevention.

In this investigation, patients were categorized based on the duration of alcohol drinking, daily drinking quantity, and cumulative alcohol intake. The overall findings revealed that individuals classified as non-drinkers or light drinkers exhibited superior PFS and OS rates compared to those categorized as moderate drinkers or heavy drinkers. Furthermore, a synergistic relationship between smoking and alcohol drinking was identified, exerting an impact on the prognosis of NPC. It is important to acknowledge certain limitations inherent in this study. Specifically, more detailed information regarding variables such as the age at which patients commenced drinking, the alcoholic strength of the beverages consumed, and the specific types of alcoholic beverages (e.g. wine and liquor) were not explicitly recorded. This lack of granularity in data collection could potentially introduce bias into the statistical results obtained. In conclusion, this study provides evidence indicating that moderate-to-heavy alcohol drinking represents a negative prognostic factor for NPC. Additionally, while smoking alone may not function as an independent prognostic factor for NPC, it can significantly influence the prognosis of NPC patients when combined with alcohol drinking.

The limitations of this study are related to its retrospective nature. We find it different to obtain the exact information about age at smoking initiation, types of Smoking, different alcohol levels, etc. These may lead to potential bias. Another possible limitation of this study is high rate of patients lost to follow-up, which may affect the statistical results, may also be the reason why smoking was not an independent prognosis factor for NPC in this cohort. So, this will be addressed by increasing the number of cases in the future.

## Conclusion

In this cohort, smoking is not an independent prognostic factor for NPC. However, alcohol drinking is a significant factor influencing the prognosis of nasopharyngeal carcinoma, with the adverse effects further amplified when combined with smoking.

## Funding and acknowledgments

This study was funded by National Natural Science Foundation (81960489, 82260485), Yunnan provincial health commission medical reserve talent training program (H-201634), Yunnan Ten Thousand People Plan “Young Top Talents” Project (YNWR-QNBJ-2019-056), General project of Yunnan basic research plan (202001AT070056), Science Research Fund Project of Yunnan Education Department in 2019 (2019J1287). We also wish to thank Dr. Zhang Ming for his statistical support.

## Declaration of competing interest

The authors declare no conflicts of interest.
